# Supplementary material for: Thiol-Based Redox Molecules: Potential Antidotes for Acrylamide Toxicity
Source: Antioxidants (Basel). 2024 Nov 21;13(12):1431. doi: 10.3390/antiox13121431 (PMC11672525; doi:10.3390/antiox13121431)
Supplement: Supplementary file 1 [file antioxidants-13-01431-s001.zip › antioxidants-3238488-supplementary.pdf]

Supporting Materials

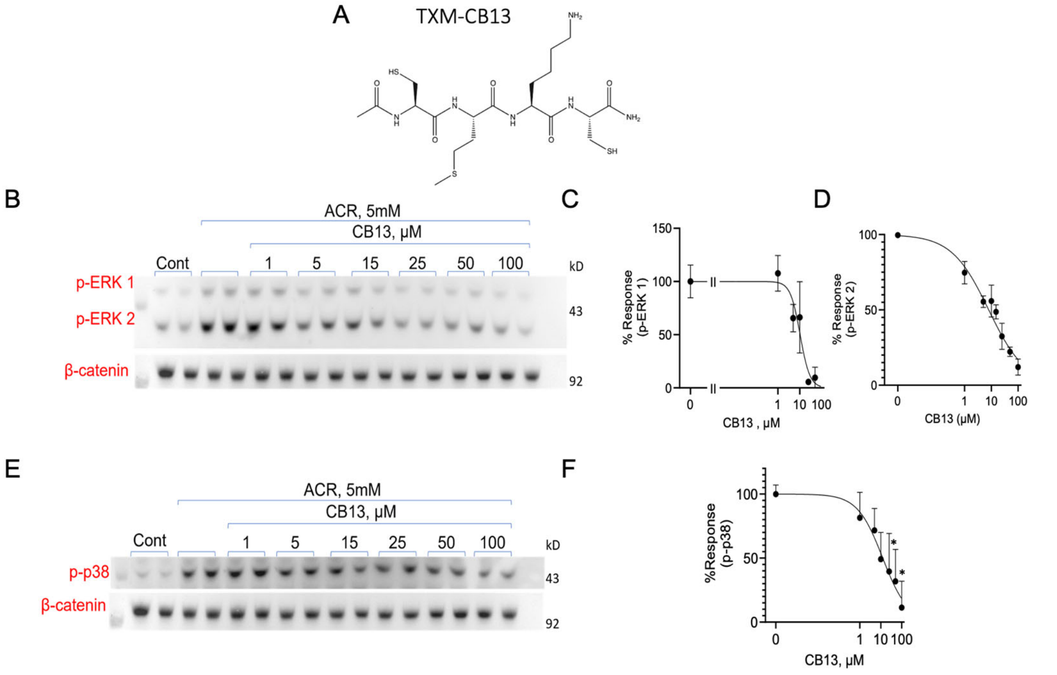

Figure S1. TXM-CB13 reverses ACR-induced activation of MAPKs. (**A**) The structure of TXM-CB13 (**B**) PC12 cells were treated with 5mM ACR for 2h, washed, and incubated with increasing concentrations of TXM-CB13. Cell lysate proteins were separated on 10% SDS-PAGE and inhibition of ACR-induced phosphorylation of (**C**) ERK1 and (**D**) ERK2 was calculated and quantified by normalization to β-catenin (**E**,**F**) p38^{MAPK} phosphorylation was visualized using the corresponding anti-phospho p38^{MAPK} antibodies and quantified by normalization to β-catenin. The values shown are averages (±SEM) of three independent experiments normalized to β-catenin the phosphorylation state of cells treated with ACR after 3 h. Student's *t*-test (two populations) was performed for ACR treated cells * $p < 0.05$.

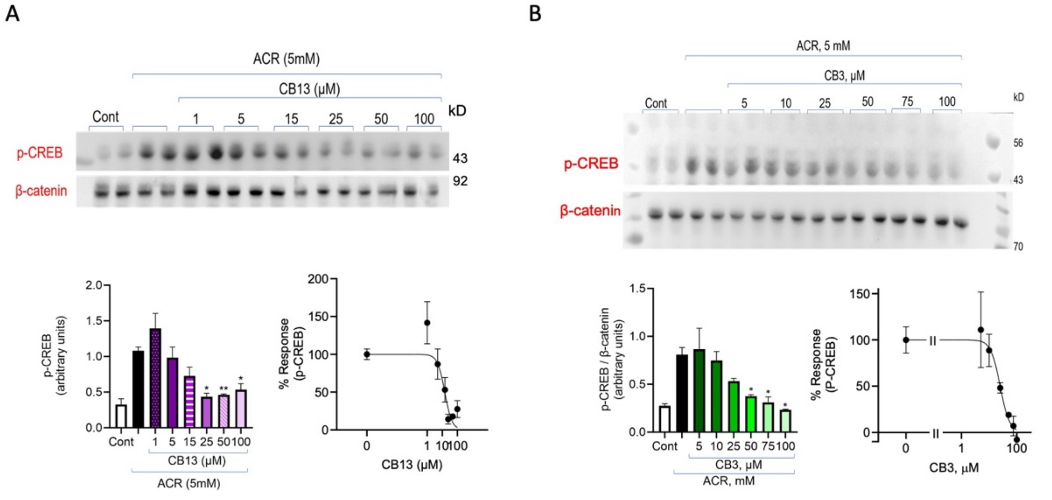

Figure S2. Inhibition of ACR-induced phosphorylation of CREB. PC12 cells were treated with 5mM ACR for 2h, washed, and with increasing concentrations of (**A**) TXM-CB13 and (B) TXM-CB3 for an additional 1 h. Cell lysate proteins were loaded in equal amounts and separated on 10% SDS-PAGE. Inhibition of ACR-induced phosphorylation of CREB was quantified and the values shown are averages (±SEM) of two independent experiments normalized to β-catenin. Student's *t*-test (two populations) was performed for ACR treated cells * $p < 0.05$; ** $p < 0.01$.
